# High and low value care recommended and undertaken prior to knee or hip arthroplasty: a survey study

**DOI:** 10.1186/s12891-023-06406-w

**Published:** 2023-04-29

**Authors:** Kathryn Mills, Anne-Marie Brewster, Danella Hackett, Chris Cheung, Michael Solomon, Justine Naylor

**Affiliations:** 1 Department of Health Sciences, Macquarie University, Sydney, Australia; 2 Southern Local Health District, New South Wales Health, Cooma, Australia; 3 South West Sydney Local Health District, Sydney, NSW Australia; 4 Sydney Hip and Knee, Sydney, Australia; 5 Whitlam Orthopaedic Research Centre, Liverpool, Australia

**Keywords:** Osteoarthritis, Non-surgical care, Quality of care, Implementation, Social determinants

## Abstract

**Introduction:**

National and international clinical practice guidelines have stratified the value of osteoarthritis (OA) interventions. Interventions with strong evidence supporting effectiveness and benefit are ‘high value care’. Appointment attendance, audits and practitioner surveys are widely used to determine frequency of recommendations and adherence to high value care. Greater patient reported data is needed in this evidence base.

**Objective:**

To describe the frequency of high and low value care being recommended and undertaken by individuals awaiting OA-related lower limb arthroplasty. To examine sociodemographic or disease-related variables associated with being recommended different levels of care.

**Methods:**

A cross-sectional survey of 339 individuals was conducted in metropolitan and regional hospitals and surgeon consultation rooms across New South Wales (NSW), Australia. Individuals attending pre-arthroplasty clinics/appointments for primary arthroplasty of the hip and/or knee were invited to participate. Respondents were asked what intervention(s) they were recommended by healthcare practitioners, or other sources of information, and what they had undertaken within two years prior to hip or knee arthroplasty. Interventions were classified as *core*, *recommended*, and *low value care* aligned with the Osteoarthritis Research Society International (OARSI) guidelines. We considered *core* and *recommended* interventions high value. The proportion of recommended and undertaken interventions were calculated. We used backwards stepwise multivariate multinomial regression to address aim three.

**Results:**

Simple analgesics were most frequently recommended (68% [95% CI 62.9 to 73.1]). 24.8% [20.2 to 29.7] of respondents were recommended high value care only. 75.2% [70.2 to 79.7] of respondents were recommended at least one *low value* intervention. More than 75% of recommended interventions were undertaken. Respondents awaiting hip arthroplasty, living outside a major city and without private health insurance had greater odds of *recommended* rather than *core* interventions being advised.

**Conclusion:**

While *high value* interventions are being recommended to individuals living with OA, in most cases they are combined with recommendations for *low value* care. This is concerning given the high rates of uptake for recommended interventions. Based on patient reported data, disease-related and sociodemographic variables influence the level of care recommended.

**Supplementary Information:**

The online version contains supplementary material available at 10.1186/s12891-023-06406-w.

## Introduction

A wide variety of interventions are available for people with knee and hip osteoarthritis (OA) prior to undergoing total arthroplasty. Multiple guidelines [1–4] provide recommendations for interventions that are suitable for everyone with OA e.g., exercise, as well as recommendations for interventions that are suitable for some patients, depending on their comorbidities. Despite guidelines, there is widespread evidence of suboptimal OA management in primary care [5–7]. A review of Australian medical records indicated that only 43% of primary care encounters resulted in a person with OA receiving appropriate care [7]. Further, non-pharmacological interventions had particularly low recommendation rates [5].

Adherence to appropriate care is often defined by counts of appointment attendance and number of referrals [8], identified through medical record audit [7] or survey of healthcare providers [5]. Patient-reported data, however, are largely missing from the literature. This is a major limitation, as quantifying or describing types of care using the care providers’ perspective or medical audits may differ from what the individual undertook. There are many reasons why an individual living with OA may choose not to undertake recommended interventions including living location [9], language barriers [6], dis-trust of their primary care practitioner [10] and a general unwilling-ness. Given people with OA largely self-manage their condition, relying on health system data for information means it is likely that current literature does not accurately represent all the interventions people are actually using.

With recent updates of national clinical practice guidelines [4] and international recommendations [2, 11], updating the prevalence of interventions being recommended to people living with knee and hip OA is needed. It is also important to understand the frequency of uptake of recommended interventions. These data will provide important insight into: whether these guidelines are making an impact; areas where greater implementation efforts are needed, and; potential patient-centred barriers to improving care.

With the above realities in mind, we conducted a survey of individuals waitlisted to undergo total knee or total hip arthroplasty (TKA and THA) to determine over the previous two-years:

1. The frequency of interventions being recommended.
2. The frequency of recommended interventions being undertaken.
3. Sociodemographic or disease-related variables associated with being recommended different types of interventions.

## Methods

### Survey development and delivery

A panel comprising researchers (n = 2), allied health clinicians (physiotherapists n = 4, exercise physiologists n = 1, clinical nurse co-ordinator n = 1)), orthopaedic surgeons (n = 2) and consumer advocates (n = 4) developed the cross-sectional survey (Appendix 1). Two consumer advocates had undergone knee replacements within the past 12 months and two were on the waiting list. The consumer advocates lived in the same areas and attended the same hospital as survey respondents. Consumer advocates piloted the survey on both tablets and desktop computers to check for technical and logistical issues. A sample of individuals (n = 5) aged 64–79 years, piloted the English version of the survey to check readability/comprehension of the questions and survey duration. The survey comprised three blocks:

- Demographic questions used to examine potential barriers and facilitators to recommended care.
- Identifying (a) clinicians other than a GP and orthopaedic surgeon that a respondent had been referred to (including a self-referral) over the past two years and (b) which interventions they had been recommended, by any healthcare practitioner or source of information over the two years leading up to their surgery. Respondents were asked to select interventions from a list including pharmacological, non-pharmacological options and gait-aids. The list was compiled based on national and international guidelines for non-surgical management of OA (whether they were recommended or not) [1–4] and experiences from clinicians and consumers on the panel.
- Identifying which interventions respondents had undertaken. This block exhibited adaptive logic such that only interventions recommended to respondents were presented.

The anonymous survey was delivered via an online platform on a tablet (Qualtrics, Seattle, USA), taking approximately 10 min to complete. The survey was single entry – partially completed responses could not be resumed. Respondents could exit the survey at any time but could not remove submitted responses. To increase culturally and linguistically diverse accessibility, the survey was translated into five languages: Arabic, Greek, Simple Chinese, Traditional Chinese and Vietnamese. These languages were chosen based on census data [12]. Translation and verification of the translated surveys were conducted by National Accreditation Authority for Translators and Interpreters (NAATI) accredited interpreters employed by NSW department of health. In cases of low literacy, respondents could choose to be assisted by a research assistant or family member, who read the survey aloud. In such cases, the research assistant remained present to ensure the family member did not complete the survey on the respondent’s behalf.

### Setting

Four metropolitan public hospitals, two regional public hospitals and two private specialist centres in NSW, Australia. The total catchment area was 700km^2^. and selected was representative of the socioeconomic characteristics and location of the Australian population [13]. Each site recruited for 6–10 consecutive weeks between January 2018 and December 2019. Eligible individuals were consecutively sampled. The study was approved by a lead ethics committee from NSW Health (South Western Sydney Local health district LNR/17/LPOOL/381) and site-specific approvals were also obtained.

### Participants

Individuals were invited to participate if they met any of the following criteria: (1) attending an arthroplasty pre-admission clinic, which typically occurs 1–2 weeks prior surgery, or (2) attending an initial appointment of the NSW publicly funded “Osteoarthritis Chronic Care Program” and on the public waitlist for a TKA or THA secondary to OA, or (3) attending an initial appointment with an orthopaedic surgeon. Only individuals undergoing primary arthroplasty were eligible. No restrictions were placed on age, number of joints being replaced, or sex. Individuals not proficient in the aforementioned languages were excluded. Eligible individuals were identified and invited to participate by medical reception staff, clinicians, or researchers. Only those interested in undertaking there were provided with a loaner iPad (Apple, California, USA), which provided a detailed explanation of the study, consent statement and survey.

### Sample size

Based on data from the Australian National Joint Replacement Registry (https://aoanjrr.sahmri.com/), there were 95,981 primary hip and knee arthroplasty surgeries in 2015, representing 4.3% of the population with OA in Australia. To ensure no more than 5% margin of error and 95% confidence level of results, 338 responses were required.

### Analysis

For aim one, recommended interventions were calculated as a proportion of the total number of responses. If a respondent was recommended multiple interventions, each intervention was tallied separately. For aim two, the number of times a recommended intervention was undertaken was calculated and reported as a proportion. To aid interpretation, we grouped interventions using current international guidelines for the non-surgical management of knee and hip OA [2] (Appendix 2): *core*, *recommended* (equivalent to Level 1 A, 1B and 2 interventions of the OARSI guidelines) and *low value care* (equivalent to levels 3–5). *Core* interventions are those identified by the guidelines as appropriate for most people with OA in nearly any scenario and are safe to be used in conjunctions with other interventions. *Recommended* interventions were those were there was at least 75% of the voting panel consulted during guideline formation agreed that the benefits of the intervention out-weigh the harms [2]. Bannuru et al. [2] identified multiple interventions that are strongly not recommended due to a lack of evidence or evidence of harm (levels 3–5). Rather than the term “not-recommended”, we adopted the term *low value care* for clarity. *Low value care* was defined by Oakes and Radomski [14] as “health service for which the harms or costs outweigh the benefits”. Modified Copper-Pearson 95% confidence intervals were calculated using the PropCI package in RStudio (Version 1.2.1335, RStudio, Inc. Boston, MA, USA).

For aim three, we allocated respondents to one of three groups (*core, recommended, low value care*) based on the highest level of intervention they received. For example, respondents indicating that they had been recommended a structured land-based exercise program (walking programs or strength-based exercise) were allocated to the *core* group regardless of other interventions recommended. Additionally, respondents’ postcodes were used to allocate Socioeconomic Indexes for Area (SEIFA) quintile and Modified Monash Model living location (major city or regional/remote). Lower SEIFA indicating worse socioeconomic disadvantage. Respondent age, education level, employment status and joint being replaced were collapsed into smaller categories for ease of data interpretation (Appendix 3).

To determine if sociodemographic variables were associated with group allocation and subsequently which interventions were recommended, multinomial regressions were conducted. Univariate models were fitted to aid interpretation of the multivariate model. A stepwise multivariate multinomial regression was then conducted using a backward stepwise procedure using p-values < 0.5 to enter the model and > 0.10 to exit the model.

## Results

There were 360 individuals invited to complete the survey, of which 339 consented to participate. Six people refused to participate prior to reading the participant information and consent form and 15 refused to participate after reading the form. This indicates a 94% response rate. Respondent characteristics are summarised in Table 1. Characteristics of individuals who refused to participate and reasons for refusal were not collected. Of the 21 individuals who declined, five (~ 24%) viewed the statement in a language other than English. Of the 339 respondents, eight (~ 2%) completed the survey in a language other than English (Arabic N = 3, Simple Chinese N = 2, Vietnamese N = 3). Forty-four respondents (11%) indicated that they would prefer to interact with the healthcare system in a language other than English.

**Table 1:** Demographics of survey responders

Variable	Frequency (N)	Proportion (%)
Sex		
Female	194	57.2
Male	145	42.8
Age		
Younger than 50 years	18	5.3
51–60 years	67	19.8
61–70 years	106	31.3
71–80 years	125	36.9
Older than 80 years	23	6.8
Country predominantly resided in		
Australia	297	85.8
Country other than Australia	48	14.2
Employment status		
Retired	152	44.8
Working (part/fulltime)	90	26.5
Government Benefit	97	28.6
Highest education level achieved		
Junior high school or below (includes no schooling)	188	55.5
Senior high school	60	17.7
Certificate or Diploma	53	15.6
University (Graduate and Post-graduate)	38	11.2
Insurance		
None	263	77.6
Hospital	76	22.4
Ancillary (e.g., physiotherapy, podiatry)	63	18.6
Residential location		
Major city	233	68.7
Regional/remote	106	31.3
Relative socioeconomic advantage/disadvantage quintile^a^		
1 (most disadvantaged)	58	17.1
2	132	38.9
3	36	10.6
4	41	12.1
5 (least disadvantage)	72	21.2
Index joint		
Hip	91	26.8
Knee	248	73.2
Years of diagnosis for index joint^b^		
Less than 1 year	68	20.1
1 to 5 years	154	45.4
6 to 10 years	68	20.1
More than 10 years	33	9.7
Previous joint replacement?		
Yes	101	29.8
No	238	70.2

a Quintiles represent relative disadvantage compared with other quintiles based on equal distribution of areas (not people). Quintiles are ranked highest to lowest disadvantage

b N = 16 (4.7%) missing responses to this question

### Recommended Interventions

**Fig. 1:**
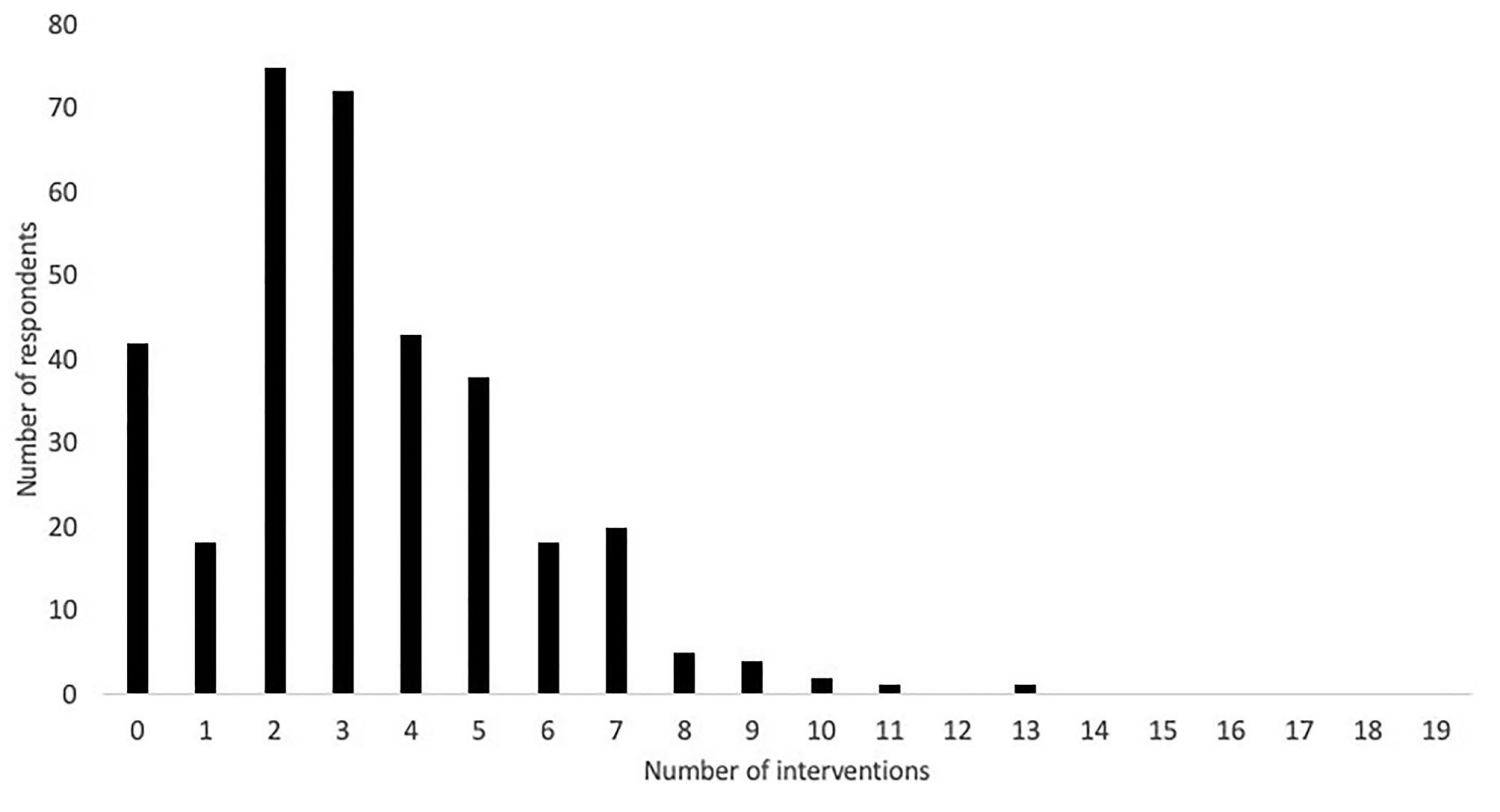
Number of interventions being recommended to respondents

**Fig. 2:**
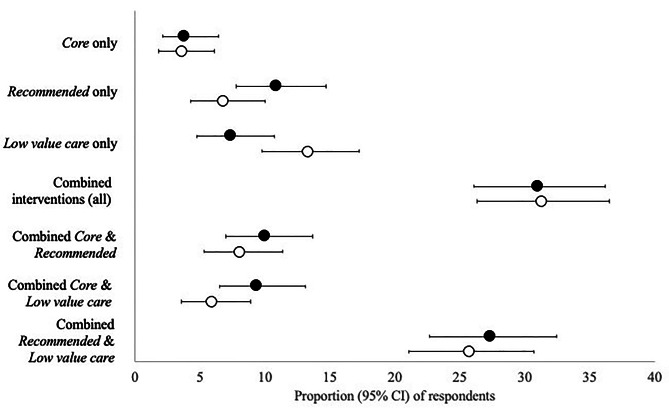
Proportion of respondent being recommended (filled circles) and undertaking (unfilled circles) different levels of care

The most frequent number of recommended interventions was 2, and the median number of interventions was 3 (Fig. 1). The most frequently recommended intervention was simple analgesics e.g., paracetamol (Table 2). For those awaiting TKA, the most frequent non-pharmacological intervention was a structured strength program (41%). For those awaiting THA, it was a walking stick (37%). Most individuals (77.9%, [73.1 to 82.2]) were recommended a combination of interventions, with 24.8% [20.2 to 29.7] of respondents receiving recommendations for only *core* or *recommended* interventions or a combination of both. Despite 54.3% [48.8 to 59.7] of respondents reporting they were recommended at least one *core* intervention, 75.2% [70.2 to 79.7] of respondents were recommended at least one *low value care* intervention. Of these, 7.4% [4.8 to 10.7] reported only being recommended *low value care* interventions (Fig. 2).

**Table 2:** Interventions that were recommended and undertaken

	Total N = 399		Knee arthroplasty		Hip arthroplasty		
Intervention	Proportion recommended (95% CI)	Proportion uptake (95% CI)	Proportion recommended (95% CI)	Proportion uptake (95% CI)	Proportion recommended (95% CI)	Proportion uptake (95% CI)	
Structured strength program	35.4 (30.3–40.7)	91.7 (85.2–95.9)	41.3 (35.1–47.7)	90.2 (82.7–95.2)	19.8 (12.2–29.4)	100 (84.5–100)	
Structured walking program	22.4 (18.1–27.2)	78.9 (68.1–84.5)	24.3 (19.1–30.1)	86.7 (75.4–94.1)	17.6 (10.4–26.9)	68.8 (41.3–88.9)	
Yoga/Pilates/mind-body	2.4 (1-4.6)	50 (15.7–84.3)	2.4 (0.8–5.2)	66.7 (22.2–95.7)	2.2 (0.2–7.7)	0.0 (0.0-84.2)	
Dietary weight management	24.5 (20-29.4)	80.7 (70.6–88.6)	26.3 (20.9–32.2)	80.0 (68.2–88.9)	18.7 (11.3–28.2)	82.4 (56.6–96.2)	
Topical NSAIDS	18 (14-22.5)	88.5 (77.8–95.2)	20.2 (15.4–25.8)	86.0 (73.2–94.2)	12.1 (6.2–18.1)	100 (71.5–100)	
Hydrotherapy	11.2 (8-15.1)	78.9 (62.7–90.4)	11.3 (7.6–15.9)	82.1 (63.1–93.9)	9.9 (4.6–17.9)	66.7 (29.9–92.5)	
Walking stick	34.5 (29.4–39.8)	82.1 (73.1–88.5)	33.6 (27.7–39.9)	81.9 (71.2–89.5)	37.4 (27.4–48.1)	82.4 (65.5–93.2)	
Crutches	7.7 (5.1–11)	84.6 (65.1–95.6)	6.9 (4.1–10.8)	94.1 (71.3–99.8)	9.9 (4.6–17.9)	66.7 (29.9–92.5)	
Knee brace/sleeve	13.6 (10.1–17.7)	78.3 (63.6–89.1)	18.2 (13.6–24)	80.0 (65.4–90.4)	1.1 (0.0-5.9)	0 (0.0-97.5)	
Walking frame	4.1 (3.3–6.8)	0 (0.0-0.2)	3.2 (1.4–6.3)	0 (0.0-3.7)	6.6 (2.4–13.8)	0 (0.0-4.6)	
Oral NSAIDS	31.6 (26.6–36.8)	91.6 (84.6–96)	34.8 (28.9–41.1)	91.9 (83.9–96.7)	23.1 (14.9–33.1)	90.5 (69.7–98.8)	
Intra-articular corticosteroid	20.4 (16.2–25)	94.2 (85.8–98.4)	21.9 (16.8–27.5)	96.3 (87.2–99.5)	16.5 (9.5–25.7)	86.7 (59.5–98.3)	
Foot orthoses/wedge	5 (2.9–7.9)	88.2 (63.5–98.5)	5.7 (3.1–9.3)	78.6 (49.2–95.3)	3.3 (0.1–9.3)	100 (29.2–100)	
Anti-epileptic medication	4.1 (2.2–6.8)	100 (76.8–100)	4 (1.9–7.3)	100 (69.1–100)	4.4 (1.2–10.9)	100 (39.8–100)	
Simple analgesics	68.1 (62.9–73.1)	94.8 (91.1–97.3)	70.9 (64.7–76.4)	94.9 (90.5–97.6)	60.4 (49.6–70.5)	94.5 (84.9–98.9)	
Weak opioid	18 (14-22.5)	95.1 (86.3–98.1)	16.6 (12.1–21.8)	100 (91.4–100)	22 (13.9–31.9)	85.0 (62.1–96.8)	
Strong opioid	9.4 (6.5–13.1)	68.8 (50.0-83.9)	8.5 (5.3–12.7)	57.1 (34-78.2)	12.1 (6.2–18.1)	90.9 (58.7–99.8)	
Arthroscope	4.4 (2.5–7.2)	93.3 (68.1–99.8)	5.7 (3.1–9.3)	92.9 (66.1–99.8)	1.1 (0.0-5.9)	0.0 (0.0-97.5)	

### Undertaken interventions

Most participants undertook interventions that had been recommended to them (Table 2). Adherence was more than 80% for pharmacological interventions, except for strong opioids, and more the 75% for non-pharmacological intervention, except for yoga/Pilates and walking frames. While 48.7% [43.2 to 54.1] of respondents undertook at least one *core* intervention, 70.2% [65 to 75] undertook at least one *low value care* intervention. 18.3% [14.3 to 22.8] of respondents only undertook *core* or *recommended* interventions, or a combination of both. Whereas 13.3% [9.8 to 17.3] of respondents only undertook *low value care* interventions (Fig. 2).

### Socioeconomic variables associated with recommended interventions

Of the 10 univariate analyses (Table 3), joint being replaced, years since diagnosis, living location and having private ancillary health insurance were included in the multivariate model (Table 4). Only associations between *core* and *recommended* interventions were significant and sufficiently powered. Individuals awaiting THA, those living in regional/remote areas and who did not have ancillary private health insurance, covering healthcare such as physiotherapy, dieticians, and exercise physiology, had between 1.75 [1.01 to 3.01] and 5.06 [2.24 to 11.45] times the odds of *recommended* interventions being advised over *core* interventions. In contrast, increasing years since diagnosis decreased the odds of *recommended* interventions being advised over *core* interventions.

**Table 3:** Univariate analyses for association between sociodemographic variables and being recommended *core* (referent) (n = 181), *recommended (+/- low value care)* (n = 129) and *low value care* only (n = 29) interventions

	*Recommended (+/- low value care)*			*Low Value Care Only*		
Variable	Odds ratio	95% CI	Sig (p-value)	Odds ratio	95% CI	Sig (p-value)
Sex						
Female	Referent			Referent		
Male	1.64	1.0, 2.59	0.035	1.35	.61, 2.98	0.457
Age						
Younger than 50 years	Referent			Referent		
51–60 years	1.67	0.53, 5.44	0.376	1.3	0.13, 12.76	0.823
61–70 years	1.34	0.43, 4.11	0.612	2.16	0.25, 18.36	0.479
71–80 years	2.12	0.7, 6.42	0.182	1.97	0.23, 16.83	0.537
Older than 80 years	2.4	0.61, 9.37	0.208	3.6	0.32, 40.23	0.298
Joint						
Knee	Referent			Referent		
Hip	3.1	1.85, 5.2	< 0.001	0.93	0.33, 2.61	0.888
Years since diagnosis						
Less than 1 year	Referent			Referent		
1 to 5 years	0.48	0.26, 0.88	0.018	0.88	0.29, 2.67	0.817
6 to 10 years	0.35	0.17, 0.73	0.005	1.04	0.31, 3.53	0.950
More than 10 years	0.33	0.15, 0.73	0.006	0.32	0.06, 1.81	0.200
Education						
Junior high school or below	Referent			Referent		
Senior high school	0.5	0.26, 0.95	0.035	0.97	0.35, 2.7	0.958
Certificate or Diploma	0.88	0.46, 1.67	0.688	1.38	0.49, 3.93	0.541
University	0.31	0.13, 0.72	0.006	0.97	0.09, 1.99,	0.279
Employment status						
Retired	Referent			Referent		
Working (part/fulltime)	0.68	0.38, 1.21	0.189	0.98	0.37, 2.54	0.961
Government Benefit	1.68	0.98, 2.88	0.060	1.5	0.58, 3.85	0.399
Socioeconomic disadvantage						
1	Referent			Referent		
2	0.95	0.5, 1.82	0.891	1.32	0.39, 4.44	0.65
3	1.43	0.58, 3.5	0.435	3.21	0.78, 13.23	0.106
4	1.25	0.54, 2.87	0.599	1.18	0.24, 5.89	0.836
5	0.45	0.21, 0.96	0.04	0.6	0.14, 2.58	0.492
Residential location						
Major city	Referent			Referent		
Regional/remote	1.74	1.07, 2.84	0.027	2.07	0.92, 4.66	0.078
Private hospital insurance						
Yes	Referent			Referent		
No	4.1	2.13, 7.88	< 0.001	1.76	0.68, 4.56	0.243
Private ancillary insurance						
Yes	Referent			Referent		
No	5.93	2.7, 13.01	< 0.001	2.45	0.81, 7.4	0.11

**Table 4:** Multivariate analysis for association between sociodemographic variables and *core* (referent) (n = 181), *recommended (+/- low value care)* (n = 129) and *low value care* only (n = 29) interventions

	*Recommended (+/- low value care)*			*Low Value Care Only*		
Variable	Odds ratio	95% CI	Sig (p-value)	Odds ratio	95% CI	Sig (p-value)
Joint						
Knee	Referent			Referent		
Hip	2.55	1.46, 4.46	0.001	0.84	0.29, 2.47	0.754
Years since diagnosis						
Less than 1 year	Referent			Referent		
1 to 5 years	0.46	0.24, 0.89	0.023	0.73	0.23, 2.33	0.591
6 to 10 years	0.52	0.23, 1.19	0.123	1.00	0.27, 3.72	0.990
More than 10 years	0.32	0.14, 0.76	0.009	0.25	0.04, 1.44	0.119
Private ancillary insurance						
Yes	Referent			Referent		
No	5.06	2.24, 11.45	< 0.001	2.59	0.82, 8.14	0.104
Residential location						
Major city	Referent			Referent		
Regional/remote	1.75	1.01, 3.01	0.045	1.95	0.83, 4.62	0.127

## Discussion

This study utilised patient-reported data to quantify interventions being recommended and undertaken prior to hip or knee OA-related arthroplasty. Over 50% of our sample reported being recommended a *core* or *recommended* intervention consistent with international guidelines [2]. This suggests some improvement since the 2014 CareTrack study [7], which reported on the proportion of primary care consultations that resulted in a recommendation for an evidence-based intervention. However, in most cases *core* interventions were recommended and undertaken in combination with a *low value care* intervention. As such, *low value care* interventions continue to be prevalent in the two years prior to arthroplasty. The odds of *recommended* interventions rather than *core* interventions being advocated increased for individuals awaiting THA, those who were not privately insured and those who lived regionally/remotely.

Aligned with previous research [5, 6], between 60 and 70% of respondents report being recommended simple analgesics. Recommending simple analgesics, such as paracetamol, is controversial as clinical practice guidelines are conflicting. In contrast to older guidelines [1, 3, 4, 11], a recent international guideline [2] and Cochrane review [15] indicate that simple analgesics are associated with little-to-no clinical efficacy and are not recommended for people with hip and knee OA. This highlights the need for clinical practice guidelines to be living documents that are updated frequently and channelled appropriately so clinicians remain aware of such major changes.

By asking respondents which interventions they had undertaken, our data indicate generally high adherence to recommended interventions. This is an important difference to previous studies [16–18] quantifying adherence as attending a facility or appointment. In some cases, self-implemented interventions, such as structured walking programs, may be more appropriate than those requiring people to attend specific appointments. There is evidence that individuals with OA, assigned to a self-directed walking group not only had equivocal adherence rates to individuals assigned to a facility-directed group, but also reported higher quality of life scores over 12-months (16). However, in our study, the proportion of people who undertook only *low value care* interventions was almost double the proportion of people who were recommended only *low value care* interventions. This indicates that a proportion of individuals who were recommended *core* or *recommended* interventions preferentially undertook *low value care*. This suggests that adopting strategies focused on self-directed walking programs as a means of increasing adherence to *core* interventions is unlikely to increase adherence. Subsequently, more research into increasing patient motivation is needed.

Several sociodemographic variables contributed to individuals not being recommended *core* interventions. This was limited to comparisons between *core* and *recommended* interventions, likely because there were too few people in the *low value care* group when stratified based on the highest level of recommended intervention. It is not surprising that individuals awaiting THA had higher odds of having *recommended* rather than *core* interventions advocated to them. A previous study examining GP-activity indicated that people with hip OA experienced lower rates of physical management and higher rates of imaging and referral to orthopaedic surgeons than people with knee OA [6]. There are several potential reasons for this. Multiple case series of individuals presenting to OA management programs have observed that people with hip OA have more severe radiological disease, worse joint pain and function scores and greater levels of distress on presentation than individuals with knee OA [19, 20]. Further, more people with hip OA live regionally/remotely than people with knee OA [6]. Combined with a scarcity of high-quality research and resources that specifically target hip OA there is little available to assist GPs’ clinical decision making when caring for people with hip OA.

A recent qualitative study identified that GPs’ knowledge on the benefit and suitability of exercise in the management of OA is inadequate, and, in some cases, inaccurate [21]. This may explain why almost 50% of respondents in this study were not recommended a *core* intervention and less than one-quarter were recommended only high value care. As *core* interventions, namely exercise, would ideally require accessing allied health services, GPs may be considering an individuals’s insurance status or access to these services when making recommendations. However, to attribute a lack of *core* intervention recommendations solely to economic or access disparity is potentially incorrect, as there were no significant differences observed in recommended care between SEIFA codes. Increasing publicly funded community-based services may therefore not be a universal solution. It is likely that there is no single solution. Multifaceted implementation strategies have greater likelihood of success [22]. In Australia, such strategies need to include increasing GP knowledge, consumer health literacy and remote healthcare delivery.

In addition to the lack of power of the *low value* care group for the sociodemographic analysis, several other study limitations need to be considered. Few respondents completed a translated survey. While it is feasible that people were comfortable completing the survey in English, it also suggests that the experiences of people from cultural and linguistic diverse backgrounds are underrepresented in this study. This study was also reliant on respondent recall, resulting in a high risk of recall bias. Due to the design of the study, this risk was unavoidable, however we used several recommended strategies [23] to minimise the risk: asking for recall over a relatively recent time frame; focusing on interventions as a whole, rather than specifics of the intervention; providing examples of interventions (e.g., medication brand names and exercise types) to serve as memory prompts. Despite these risk minimisation strategies, it is still possible that interventions that had little clinical efficacy or resulted in minimal interruption to a respondent’s daily routine were more likely effected by recall bias as the accuracy of memory of a certain event is usually dependent on the impact of that event [23]. Last, despite osteoarthritis education being widely acknowledged as a *core* intervention, it was not included as a response option in our survey. Pilot testing showed a high level of contentions amongst consumer advocates and health professionals regarding what constitutes ‘education’. To avoid confusion within our sample, the development group elected to remove education as a response option. This risks participants who did receive a *core* management not being acknowledged, but also reflects with potential variation in quality, and subsequent usefulness, of this education.

## Conclusion

Patient-reported accounts indicate that almost half are recommended internationally recognised core interventions for the management of their hip or knee OA, and most will undertake what is recommended. However, recommendations and uptake of *low value care* interventions remain high. This suggests that a major barrier to individuals participating in high value care for OA prior to arthroplasty is that such interventions are not being recommended to them in primary care. We have identified that index joint, living location and insurance status may influence intervention recommendations. In doing so, we have highlighted that more research and policy are needed pertaining to implementation of interventions, particularly for those with hip OA.

## Electronic supplementary material

Below is the link to the electronic supplementary material.

Supplementary Material 1

Supplementary Material 2

Supplementary Material 3

## Data Availability

The dataset generated are not publicly available as participants provided specific consent (data only to be used for the purposes of this study), but may be made available from the corresponding author on reasonable request.

## Abbreviations

OA: Osteoarthritis
TKA: Total Knee Arthroplasty
THA: Total hip Arthroplasty
GP: General Practitioner
NSW: New South Wales
SEIFA: Socioeconomic Indexes for Area

## References

[1] NICE. : Osteoarthritis: Care and Management in Adults. In. Edited by Excellence NIfHaC. London: National Institute for Health and Care Excellence (UK); 2014. 25340227

[2] Bannuru RR, Osani MC, Vaysbrot EE, Arden NK, Bennell K, Bierma-Zeinstra SMA, Kraus VB, Lohmander LS, Abbott JH, Bhandari M (2019). OARSI guidelines for the non-surgical management of knee, hip, and polyarticular osteoarthritis. Osteoarthritis Cartilage.

[3] Jevsevar DS. Treatment of osteoarthritis of the knee: evidence-based guideline, 2nd edition. *JAAOS - Journal of the American Academy of Orthopaedic Surgeons* 2013, 21(9):571–576. 10.5435/JAAOS-21-09-571 23996988

[4] RACGP (2018). Guideline for the management of knee and hip osteoarthritis.

[5] Basedow M, Williams H, Shanahan EM, Runciman WB, Esterman A. Australian GP management of osteoarthritis following the release of the RACGP guideline for the non-surgical management of hip and knee osteoarthritis. BMC Research Notes 2019:1–8. 10.1186/s13104-015-1531-z PMC4593191 26438323

[6] Brand CA, Harrison C, Tropea J, Hinman RS, Britt H, Bennell K (2014). Management of Osteoarthritis in General Practice in Australia. Arthritis Care Res.

[7] Runciman WB, Hunt TD, Hannaford NA, Hibbert PD, Westbrook JI, Coiera EW, Day RO, Hindmarsh DM, McGlynn EA, Braithwaite J (2012). CareTrack: assessing the appropriateness of health care delivery in Australia. Med J Australia.

[8] Brand CA, Ackerman IN, Tropea J (2014). Chronic disease management: improving care for people with osteoarthritis. Best Pract Res Clin Rheumatol.

[9] Thomas SL, Wakerman J, Humphreys JS. Ensuring equity of access to primary health care in rural and remote Australia - what core services should be locally available? International Journal for Equity in Health 2015:1–8. 10.1186/s12939-015-0228-1 PMC4625941 26510998

[10] Bowden JL, Lamberts R, Hunter DJ, Melo LR, Mills K (2020). Community-based online survey on seeking care and information for lower limb pain and injury in Australia: an observational study. Bmj Open.

[11] McAlindon TE, Bannuru RR, Sullivan MC, Arden NK, Berenbaum F, Bierma-Zeinstra SM, Hawker GA, Henrotin Y, Hunter DJ, Kawaguchi H (2014). OARSI guidelines for the non-surgical management of knee osteoarthritis. Osteoarthr Cartil.

[12] ABS. Main language other than English spoken at home. In. Edited by Statistics ABo; 2016.

[13] ABS. Census of Population and House: Reflective Australia-Stories from the Census. In. Edited by Statistics ABo; 2016.

[14] Oakes AH, Radomski TR (2021). Reducing low-value care and improving Health Care Value. JAMA.

[15] Leopoldino AO, Machado GC, Ferreira PH, Pinheiro MB, Day R, McLachlan AJ, Hunter DJ, Ferreira ML (2019). Paracetamol versus placebo for knee and hip osteoarthritis. Cochrane Database Syst Rev.

[16] Brand CA, Amatya B, Gordon B, Tosti T, Gorelik A (2010). Redesigning care for chronic conditions: improving hospital-based ambulatory care for people with osteoarthritis of the hip and knee. Intern Med J.

[17] Brosseau L, Wells GA, Kenny GP, Reid R, Maetzel A, Tugwell P, Huijbregts M, McCullough C, Angelis GD, Chen L (2012). The implementation of a community-based aerobic walking program for mild to moderate knee osteoarthritis (OA): a knowledge translation (KT) randomized controlled trial (RCT): part I: the Uptake of the Ottawa Panel clinical practice guidelines (CPGs). BMC Public Health.

[18] Hoogeboom TJ, Kwakkenbos L, Rietveld L, Broeder, AAd (2012). Bie RAd, Ende CHMvd: feasibility and potential effectiveness of a non-pharmacological multidisciplinary care programme for persons with generalised osteoarthritis: a randomised, multiple-baseline single-case study. Bmj Open.

[19] Le Marshall KF, Yee B, Dieppe PA, Leung A, Page C, Choong PF, Dowsey M, Lim KK. Differences between patients with hip and knee osteoarthritis. Osteoarthritis and Cartilage 2013,21.

[20] Dabare C, Marshall KL, Leung A, Page C, Choong P, Lim K (2017). Difference in presentation, progression and rates of arthoplasty between hip and knee osteoarthritis: Observation from an osteoarthritis cohort study - a clear role for conservative management. Int J Rheum Dis.

[21] Egerton T, Nelligan RK, Setchell J, Atkins L, Bennell KL (2018). General practitioners’ views on managing knee osteoarthritis: a thematic analysis of factors influencing clinical practice guideline implementation in primary care. Bmc Rheumatol.

[22] Brosseau L, Wells GA, Kenny GP, Reid R, Maetzel A, Tugwell P, Huijbregts M, McCullough C, Angelis GD, Chen L (2012). The implementation of a community-based aerobic walking program for mild to moderate knee osteoarthritis: a knowledge translation randomized controlled trial: part II: clinical outcomes. BMC Public Health.

[23] Talari K, Goyal M (2020). Retrospective studies – utility and caveats. J Royal Coll Physicians Edinb.

