# Supplementary material for: High and low value care recommended and undertaken prior to knee or hip arthroplasty: a survey study
Source: BMC Musculoskelet Disord. 2023 Apr 29;24:337. doi: 10.1186/s12891-023-06406-w (PMC10148453; doi:10.1186/s12891-023-06406-w)
Supplement: Supplementary file 1 — Supplementary Material 1 [file 12891_2023_6406_MOESM1_ESM.docx]

Appendix 1

Treatment Options prior to Total Arthroplasty Survey (TOTAS)

1. What sex do you identify as?

- Female
- Male
- Other

1. How old are you

- 30-40 years
- 41-50 years
- 51-60 years
- 61-70 years
- 71-80 years
- 81-90 years

1. Which country have you lived for most of your life?

*Open field question*

1. Do you identify as Aboriginal or Torres Strait Islander?

- Yes
- No

1. Which language would you prefer your healthcare provider to speak to you in?

*Open field question*

1. Which of the following best describes your current employment status?

- Working full time
- Working part time
- Retired
- Unemployed
- Government Benefits/Pension
- I would prefer not to answer this question

1. What is the postcode for where you live?

*Open field question*

1. What is the highest level of education you have completed?

- No schooling
- Primary/elementary school only
- Year 11 or below
- Year 12 (Final year of high school/college)
- Certificate III of IV
- Diploma/Advanced Diploma
- University undergraduate degree
- Postgraduate degree

1. Tick any of the following that you have private health insurance for:

- Physiotherapy
- Chiropractors
- Podiatry
- Natural therapies
- Dietician
- Psychology
- Private Hospital
- Exercise physiologist
- Other? *(please specify)*
- I do not have private health insurance

1. Which joint are you scheduled to get replaced in THIS surgery (*select all that apply)*

- Left hip
- Right hip
- Left knee
- Right knee

1. When were you diagnosed with osteoarthritis by a doctor for the joint you are scheduled to have replaced in THIS surgery?

- Less than 1 year ago
- 1-5 years ago
- 6-10 years ago
- 11-15 years ago
- 15-20 years ago
- >20 years ago

1. Have you had any other knee or hip joints replaced? (tick all that apply)

- No
- Left hip
- Right hip
- Left knee
- Right knee

1. Which of the following practitioners have you seen prior to attending this clinic for the joint you have elected to have this surgery on in the past 2 years? (tick all that apply)

- General practitioner (GP)
- Physiotherapist
- Dietitian
- Podiatrist
- Exercise Physiologist
- Psychologist
- Social Workers
- Occupation Therapist
- Rheumatologist
- Orthopaedic Surgeon
- Other *(please specify)*

1. What treatments were you **offered or recommended** in the past 2 years prior to undertaking this surgery? (tick all that apply)

- Strength-based exercises (individual or group classes)
- Weight loss
- Walking program
- Hydrotherapy
- Yoga
- Pilates
- Oral non-steroidal medication such as mobic or celebrex
- Topical anti-inflammatories such as voltaren gel or fisocream
- Weak opioids such as codeine, hydrocodone plus acetaminophen, and tramadol
- Strong opioids such as oxycodone, hydromorphone, morphine, fentanyl, and oxymorphone.
- Simple analgesics such as Panadol osteo or panadol
- Anti-epileptic drug such as pregabalin (lyrica)
- Pain-management programs
- Arthroscopic surgery, that is a knee or hip arthroscope,
- Cortisone injection into the joint
- Platelet rich plasma injections
- Synvisc (hylan injection)
- Stem cell therapy
- Other *(please specify)*
- None of the above

1. *(Logic: Skip this question if None of the above from question 13 was chosen)* Which of these treatments did you choose to **undertake**?

- Strength-based exercises (individual or group classes)
- Weight loss
- Walking program
- Hydrotherapy
- Yoga
- Pilates
- Oral non-steroidal medication such as mobic or celebrex
- Topical anti-inflammatories such as voltaren gel or fisocream
- Weak opioids such as codeine, hydrocodone plus acetaminophen, and tramadol
- Strong opioids such as oxycodone, hydromorphone, morphine, fentanyl, and oxymorphone.
- Simple analgesics such as Panadol osteo or panadol
- Anti-epileptic drug such as pregabalin (lyrica)
- Pain-management programs
- Arthroscopic surgery, that is a knee or hip arthroscope,
- Cortisone injection into the joint
- Platelet rich plasma injections
- Synvisc (hylan injection)
- Stem cell therapy
- Other *(please specify)*
- None of the above

1. Have you been **offered or recommended** any of the following devices or interventions in the past 2 years prior to undertaking this surgery?

- Walking stick
- Crutches
- Orthotics for your shoes
- Knee braces/sleeves
- Retraining your walk (e.g. walk with your feet turned out)
- Other *(please specify)*
- *None of the above*

1. *(Logic question: skip if None of the above was chosen in question 16)* Which device did you **choose to use**?

- Walking stick
- Crutches
- Orthotics for your shoes
- Knee braces/sleeves
- Retraining your walk (e.g. walk with your feet turned out)
- None of the above

1. Did you seek out any additional treatments on your own?

- Yes
- No
- Don’t know

1. *(Logic question: Only open if “yes” was ticked in question 18)* What treatments additional treatments did you undertake?

*Open ended question*

1. *(Logic question: only open if “no” was ticked in question 18)*

Would you have liked to have been offered another form of treatment prior to surgery?

- Yes
- No
- Don’t know

1. *(Logic: only open if “yes” was ticked in question 20)*

Please tick any other treatment and/or devices you would have been interested in being recommended

- Strength-based exercises (individual or group classes)
- Weight loss
- Walking program
- Hydrotherapy
- Yoga
- Pilates
- Oral non-steroidal medication such as mobic or celebrex
- Topical anti-inflammatories such as voltaren gel or fisocream
- Weak opioids such as codeine, hydrocodone plus acetaminophen, and tramadol
- Strong opioids such as oxycodone, hydromorphone, morphine, fentanyl, and oxymorphone.
- Simple analgesics such as Panadol osteo or panadol
- Anti-epileptic drug such as pregabalin (lyrica)
- Pain-management programs
- Arthroscopic surgery, that is a knee or hip arthroscope,
- Cortisone injection into the joint
- Platelet rich plasma injections
- Synvisc (hylan injection)
- Stem cell therapy
- Alternative therapies (e.g., Chondroitin or Glucosamine)
- Alternative approaches (e.g., Acupuncture, massage)
- Walking stick
- Crutches
- Orthotics for your shoes
- Knee braces/sleeves
- Retraining your walk (e.g. walk with your feet turned out)
- I do not know what other treatments I would like to have been offered
- Other *(please specify)*

All done. Thank you for taking time to complete this survey. Your responses will go towards improving the information and options available for other people with joint pain. We could not improve without you!
