# Supplementary material for: High and low value care recommended and undertaken prior to knee or hip arthroplasty: a survey study
Source: BMC Musculoskelet Disord. 2023 Apr 29;24:337. doi: 10.1186/s12891-023-06406-w (PMC10148453; doi:10.1186/s12891-023-06406-w)
Supplement: Supplementary file 2 — Supplementary Material 2 [file 12891_2023_6406_MOESM2_ESM.docx]

Appendix 2: Interventions collapsed for analysis. *Core*, *recommended* and *low value care* classifications are adopted from Bannuru et al., (2), OARSI guidelines for non-surgical management of knee, hip and polyarticular osteoarthritis (OA)

| Intervention categories | Interventions included in category |
| --- | --- |
| Core | Strength-based exercises (individual or group classes)  Weight loss^a^  Walking program  Yoga/Pilates^b^ |
| Recommended | Topical anti-inflammatories such as voltaren gel or fisocream  Oral non-steroidal medication such as mobic or celebrex  Pain-management programs  Cortisone injection into the joint  Platelet rich plasma injections  Synvisc (hylan injection)  Hydrotherapy  Walking stick  Crutches |
| Low Value Care | Weak opioids such as codeine, hydrocodone plus acetaminophen, and tramadol  Strong opioids such as oxycodone, hydromorphone, morphine, fentanyl, and oxymorphone.  Simple analgesics such as Panadol osteo or Panadol  Orthotics for your shoes |

^a^ in people with hip OA, weight loss is a good clinical practice statement.

^b^ Yoga/Pilates/Mind-and-body is non-core recommended in people with hip OA
