# Supplementary material for: High and low value care recommended and undertaken prior to knee or hip arthroplasty: a survey study
Source: BMC Musculoskelet Disord. 2023 Apr 29;24:337. doi: 10.1186/s12891-023-06406-w (PMC10148453; doi:10.1186/s12891-023-06406-w)
Supplement: Supplementary file 3 — Supplementary Material 3 [file 12891_2023_6406_MOESM3_ESM.docx]

Appendix 3: Sociodemographic variables collapsed for analysis.

| Variable | Data format at collection | Data format for analysis |
| --- | --- | --- |
| Age | 30-40 years  41-50 years  51-60 years  61-70 years  71-80 years  81-90 years | Younger than 50 years  51-60 years  61-70 years  71-80 years  Older than 80 years |
| Employment status | Working full time  Working part time  Retired  Unemployed  Government benefits/pension | Working  Retired  Government benefits |
| Education | No schooling  Primary/elementary school  Year 11 or below  Year 12 (final year of high school)  Certificate III or IV  Diploma/Advanced diploma  University undergraduate degree  Postgraduate degree | Did not complete secondary  Completed secondary school  Certificate/Diploma/Advanced diploma  University degree |
| Private health insurance | Private Hospital  Physiotherapy  Chiropractors  Podiatry  Natural therapies  Dietician  Psychology  Exercise physiologist  Other  I do not have private health insurance | Private Hospital  Ancillary cover  None |
| Joint being replaced | Left hip  Right hip  Left knee  Right knee | Hip  Knee |
| Diagnosis of OA | Less than 1 year ago  1-5 years ago  6-10 years ago  11-15 years ago  15-20 years ago  >20 years ago | Less than 1 years ago  1-5 years ago  6-10 years ago  More than 10 years ago |
